# A scoring matrix approach to detecting miRNA target sites

**DOI:** 10.1186/1748-7188-3-3

**Published:** 2008-03-31

**Authors:** Simon Moxon, Vincent Moulton, Jan T Kim

**Affiliations:** 1 School of Computing Sciences, University of East Anglia, Norwich, NR4 7TJ, UK

## Abstract

**Background:**

Experimental identification of microRNA (miRNA) targets is a difficult and time consuming process. As a consequence several computational prediction methods have been devised in order to predict targets for follow up experimental validation. Current computational target prediction methods use only the miRNA sequence as input. With an increasing number of experimentally validated targets becoming available, utilising this additional information in the search for further targets may help to improve the specificity of computational methods for target site prediction.

**Results:**

We introduce a generic target prediction method, the Stacking Binding Matrix (*SBM*) that uses both information about the miRNA as well as experimentally validated target sequences in the search for candidate target sequences. We demonstrate the utility of our method by applying it to both animal and plant data sets and compare it with miRanda, a commonly used target prediction method.

**Conclusion:**

We show that *SBM *can be applied to target prediction in both plants and animals and performs well in terms of sensitivity and specificity. Open source code implementing the *SBM *method, together with documentation and examples are freely available for download from the address in the Availability and Requirements section.

## Background

microRNAs (miRNAs) are small non-coding RNAs of around 21 nt in length, which are currently receiving a great deal of attention [1,2]. They are derived from a precursor RNA hairpin structure by RNAse III-like enzymes [3], and are incorporated into the RNA induced silencing complex (RISC). Via this complex, the microRNA guides either the cleavage or translational repression of messenger RNAs (mRNAs) by binding to a region of the mRNA known as the target site [4]. In this way, miRNAs regulate a variety of cellular and molecular functions [5,6], playing important roles in, for example, organism growth and development [7,8].

New miRNAs are being discovered at an increasingly rapid rate [9]. Since they play an important role in eukaryotic gene regulation, the problem of determining their function is thus of utmost importance. Accordingly, several computational methods have been developed for miRNA target prediction – see e.g. [10-13]. These methods usually rely on finding target sequences based on a single miRNA input, and employ nucleotide complementarity and minimum free energy (MFE) calculations to identify candidate miRNA/target duplexes. Although these methods have been successfully used in target prediction e.g. [14], their specificity can be limited, i.e. they may produce many false positives [15].

Various methods have been proposed to improve the specificity of miRNA target prediction methods. For example, comparative genomics has been used to focus on sites that are conserved between species [14]. Here we concentrate on an alternative approach, the Stacking Binding Matrix (*SBM*), in which we can incorporate all of the known targets for a given miRNA (in general a miRNA may target several sites) into a search for additional targets. The number of experimentally validated miRNA targets is steadily growing, and as this number increases so too should the usefulness of the *SBM *method.

Our approach is an adaptation of the binding matrix (BM) technique for transcription factor binding site classification [16], a method that was designed to systematically maximise specificity in searches for transcription factor binding sites. In contrast to computation of the BM, which uses single nucleotide information and results in a 4 × *l *matrix for scoring words of length *l*, the *SBM *is a 16 × (*l *- 1) matrix based on dinucleotides (i.e. consecutive pairs of nucleotides). In this way, it is possible to incorporate the fundamental principle of RNA stacking energies [17] which is commonly used in miRNA detection.

## Methods

In brief, the *SBM *is computed from a multiple sequence alignment consisting of the reverse complement of the miRNA in question together with any known target sequences. The resulting matrix (or set of matrices in case the alignment contains gaps) is then used to scan and score a set of potential target sequences. Sequences having a score exceeding a user-defined threshold are returned as potential targets.

### Scoring matrices and the Binding Matrix

A *scoring matrix *for nucleotide words of length *l *is an {*A*, *C*, *G*, *U*} × *l *matrix *M *= (*m*_*bk*_). Given a word *w *= *w*[1] *w*[2] ... *w*[*l*] in the alphabet {*A*, *C*, *G*, *U*} its score *S*(*w*) is the sum of the matrix elements "selected" by the symbols in the word, that is,

$$S(w)=\sum_{k=1}^{l}m_{w[k],k}.$$

Given a threshold *S*_min_, a word *w *is classified as a *binding word *if *S*(*w*) ≥ *S*_min _and otherwise it is classified as a non-binding word. Generally, the threshold can be used to adjust sensitivity and specificity of classification: Assuming a positive correlation between density of true positives and score, lowering the threshold increases sensitivity and decreases specificity. Also, notice that for any *λ *> 0, scoring a word with the matrix *λ M *and using the threshold *λ S*_min _results in the same classification. A matrix classifier is called *consistent *with a set *B *= {*b*_1_, ..., *b*_*N*_}, of known binding words if it classifies them all correctly [18], i.e. if *S*(*b*) ≥ *S*_min _for all *b *∈ *B*. There are various ways of constructing a scoring matrix from a set of binding words [19]. The *Binding Matrix (BM) *is defined to be the matrix for which the number of words classified as binding words is minimal, under the condition that it is consistent. A method for computing the BM and a discussion of its properties is given in [16].

### Incorporating stacking into binding matrix computations

A key feature in RNA structure prediction is the incorporation of stacking energies [17]. So as to capture information from both nucleotide complementarity and base pair stacking energies, in the computation of the *SBM *we score *dinucleotides*. Formally, for nucleotide words of length *l*, *SBM *is a {*A*, *C*, *G*, *U*}^2 ^× (*l *- 1) matrix. It is computed by first converting each word *w *into the sequence *w*[1]*w*[2], *w*[2]*w*[3], ..., *w*[*l *- 1]*w*[*l*] of dinucleotides in {*A*, *C*, *G*, *U*}^2 ^and then optimising as with the BM. For performance reasons, to compute the *SBM *we use the optimisation approach described in [20] rather than the quadratic programming technique used in [16]. All *SBM*s are scaled so that a threshold of 1 corresponds to the most specific consistent classifier.

Note that in contrast to transcription factors, where only binding site sequences (binding words) are available, the reverse complement of the miRNA sequence itself provides information about the accepted target site sequences. Thus we include the reverse complement of the miRNA within the alignment of the known target sites.

### Incorporating gaps

**Figure 1:**
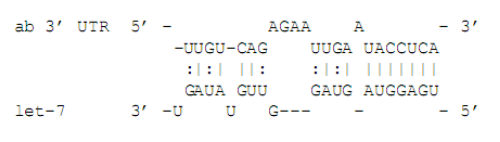
Alignment of the *Drosophila melanogaster *let-7 miRNA to a cognate target site in the 3' UTR of the *ab *gene adapted from [21, Fig. 1].

The complementarity of a miRNA binding to a target site is usually imperfect and commonly involves bulges (see Figure 1), which results in gapped alignments.

However, in common with scoring matrix-based classification approaches, the *SBM *cannot accommodate gaps directly. To address this, we employ a *set *of *SBM*s rather than a single *SBM*.

For *N *= {*A*, *C*, *G*, *U*}, let *A *= {*S*_1_, *S*_2_, ..., *S*_*n*_} denote an alignment consisting of (possibly) gapped sequences over *N *of length *l*. Denote the gap character by -, and let *s*_*i*,*j *_be the *j*-th symbol of *S*_*i*_. Suppose that *D *⊆ {1, 2, ..., *l*}. Given a sequence *S*_*i *_∈ *A*, let $S_{i}^{D}=s_{i,j_{1}}s_{i,j_{2}}...s_{i,j_{l-\left|D\right|}}$ denote the subsequence of *S*_*i *_with *j*_*k *_<*j*_*k*+1 _and *j*_*k *_∈ {1, 2, ..., *l*} - *D*, and define the *subsequence alignment *of *A *corresponding to *D *to be $A^{D}=\{S_{1}^{D},S_{2}^{D},...,S_{n}^{D}\}$ (i.e. the alignment obtained from *A *by removing the columns indexed by elements of *D*). The *gap pattern of a sequence S*_*i *_∈ *A*, denoted *G*(*S*_*i*_), is the set *G*(*S*_*i*_) = {*j *: *s*_*i*,*j *_= -}. In particular, for each *S*_*i *_∈ *A *the ungapped sequence corresponding to *S*_*i *_equals $S_{i}^{G(S_{i})}$. Correspondingly, the *gap pattern of A *is defined as *G*(*A*) = ∪_*i *_*G*(*S*_*i*_), i.e. the set of indices of those columns in *A *that contain at least one gap.

Now, let D be a subset of 2^*G*(*A*) ^(in practice we take either $D_{\text{all}}$ = 2^*G*(*A*) ^or $D_{\text{observed}}$ = {*G*(*S*) : *S *∈ *A*}). For each of the alignments $A(D)=\{A^{D}:D\in D\}$ we calculate a *SBM*. In case an alignment *A' *in A contains some gaps, each sequence *S *in *A' *that contains gaps is replaced by the set of all sequences obtained by replacing the gaps in *S *with all possible nucleotide symbol combinations (or the set of nucleotides actually observed at the gap containing position).

Once the set of *SBM*s has been computed for each alignment in A(D), query sequences are then scanned with each of the matrices, and the final score at a given base in a query sequence is taken to be the largest of the scores attained by the individual *SBM*s. As usual, a target site is predicted in case the final score exceeds a user-defined threshold. This extension to gapped alignments allows the detection of target sites with varying lengths whilst preserving specificity and consistency, both of which are key features of the original BM approach. Note that consistency is ensured since, for each sequence *S*_*i *_∈ *A*, we have *G*(*S*_*i*_) ∈ D as one alignment in A(D) must contain $S_{i}^{G(S_{i})}$. Computing *SBM*s based on $D_{\text{observed}}$ makes most use of the gap information contained in the alignment. As an alternative, computing a (larger) *SBM *set based on $D_{\text{all}}$ may allow detection of target sites that are recognised by a pairing structure different from those formed by the target sites known so far, which may be used to improve sensitivity.

### Computational complexity

The number of alignments in the set A(D) used in the calculation of *SBM *set is of order 2^|*G*(*A*)|^, and so grows exponentially with the number of columns in *A *containing gaps. Hence, our approach will not scale to long alignments containing many gaps. Even so, in practice we have found the approach to be applicable to miRNA target prediction, since usually |*G*(*A*)| ≤ 6 (as miRNAs are about 21 nt in length), resulting in at most 2^6 ^= 64 alignments in A(D). Obviously, choosing $D=D_{\text{observed}}$ rather than $D=D_{\text{all}}$ can considerably reduce |D|, particularly if gaps occur in only a few distinct patterns. Likewise, the number of alignments obtained after the gap filling procedure is performed also grows exponentially, although the approach is still feasible for miRNA targets, again due to their short length.

### Implementation

We have implemented our method in Python [22] and R [23]. The code, together with documentation and examples, is freely available for download (see Availability and Requirements).

## Results

To demonstrate the utility of the *SBM *method, we present an application to the problem of miRNA target detection for nematode worm (*Caenorhabditis elegans*), fruit fly (*Drosophila melanogaster*), mouse (*Mus musculus*), human (*Homo sapiens*) and thale cress (*Arabidopsis thaliana*). We also present a leave one out analysis, and a comparison with miRanda [10], a commonly used miRNA target prediction algorithm.

### Data

We extracted *C. elegans*, *D. melanogaster*, *M. musculus *and *H. sapiens *miRNA entries from the miRBase database, release 9.1 [9] that had more than one unique, experimentally validated target in the TarBase database [24]. The reverse complement of each miRNA was then aligned with its validated target regions using the ClustalW alignment package [25]. If local alignment algorithms are used, terminal gaps carry much less significance than internal gaps. Therefore, alignments were trimmed by removing columns containing terminal gaps at the 5' or 3' end.

*SBM *sets were computed for these alignments as described in the Methods section. The *SBM *sets were used to search for potential new targets in the UTR sequence sets obtained from BioMart [26] (see Table 1 for details). To test the applicability of the method to plant target prediction, we took a selection of *A. thaliana *miRNAs from miRBase together with validated target regions from the the *Arabidopsis *Small RNA Project Database (ASRP) [27], aligned these sequences with ClustalW, and computed *SBM*s.

**Table 1:** Summary of UTR datasets

**Organism**	**No. Sequences**	**Sequence type**	**No. Nucleotides**
*C. elegans*	12,172	UTR	2,724,326
*D. melanogaster*	11,277	UTR	4,612,168
*M. musculus*	20,271	UTR	20,009,781
*H. sapiens*	27,685	UTR	30,673,888
*A. thaliana*	31,527	cDNA	46,447,255

"No. sequences" gives total number of unique sequences in this dataset; "Sequence type" gives the sequence type used (UTR or cDNA); "No. nucleotides" gives total number of nucleotides in the UTR set.

### Summary of *SBM *Scan

On the animal data sets, we determined for each of the *SBM *sets the number of predicted targets obtained by scanning the UTR data set, using a score threshold of 1 [see Additional file 3]. As in [16], we used the number of predicted targets obtained with a consistent classifier as an indicator of the classifier's specificity.

Plant miRNA targets usually occur in the protein coding region of genes and therefore we searched the gene sequence set TAIR6_cdna_20051108 obtained from The *Arabidopsis *Information Resource (TAIR) [28] again using a threshold of 1. A summary of these results can be seen in Table 2 with full datasets available in the supplementary materials [see Additional file 1].

**Table 2:** *SBM *scan summary obtained using a score threshold of 1

**Organism**	**miRNA**	**Validated targets**	**Recovered targets**	**Potential novel targets**
*C. elegans*	*cel-miR-273*	2	2	0
*C. elegans*	*cel-let-7*	15	15	1708
*C. elegans*	*cel-miR-84*	7	7	123
*D. melanogaster*	*dme-miR-11*	4	4	0
*D. melanogaster*	*dme-miR-2*	4	4	0
*D. melanogaster*	*dme-miR-4*	8	8	23
*D. melanogaster*	*dme-miR-7*	15	15	28
*M. musculus*	*mmu-miR-124*	3	3	0
*M. musculus*	*mmu-miR-206*	3	3	0
*H. sapiens*	*hsa-miR-1*	4	4	0
*H. sapiens*	*hsa-miR-122*	3	3	0
*A. thaliana*	*ath-miR-163*	5	5	0
*A. thaliana*	*ath-miR-172*	6	6	0
*A. thaliana*	*ath-miR-390*	1	1	0
*A. thaliana*	*ath-miR-398*	2	2	0
*A. thaliana*	*ath-miR-408*	2	3	1

"miRNA" gives miRBase miRNA identifier; "Validated targets" gives number of unique validated targets present in the starting alignment; "Recovered targets" gives number of validated targets in the input alignment that were recovered; "Predicted novel targets" gives number of candidate target sequences (other than the validated targets) predicted by the *SBM *method.

In accordance with the definition of the *SBM *method, in Table 2 we see that all validated targets present in the input alignment are recovered in the scan output using a threshold of 1. In many cases no additional candidate targets are predicted using this stringent threshold, especially when there are few sequences provided in the input *SBM *set. Larger sets of validated targets tended to result in the prediction of more new candidate target sites, as illustrated in Table 2 by the cases of *dme-miR-4*, *dme-miR-7*, *cel-let-7 *and *cel-miR-84*. This reflects the consistency criterion built into the binding matrix definition; a larger input set of sequences generally tended to reduce the stringency of the classifier.

*cel-let-7 *returned 1708 predicted targets at threshold 1 which appears to be relatively high compared with the other results, but given the size the searched database (2,274,326 nt) it is a small proportion of all possible target regions. A possible reason for the large number of predicted targets is that the input sequence set used to build the *SBM *set was misaligned by ClustalW. The validated targets used to create the alignment showed a greater degree of heterogeneity that those in other alignments. Another possible explanation is that *cel-let-7 *is known to have several paralogs (*cel-miR-84*, *cel-miR-48 *and *cel-miR-241*) [29] and therefore its targets are likely to overlap with other members of this miRNA family. It has also been suggested that some miRNAs may target thousands of different genes [30] making it possible that many of the targets predicted are in fact true positives.

### Leave one out analysis

While the *SBM *method used with *S*_min _= 1 recovers all targets that are present in the input alignment, unknown targets that receive a score below 1 are likely to exist. It is possible to detect such sequences using the *SBM *method by lowering the threshold. This increases the classifier's sensitivity at the expense of reducing its specificity. To assess this effect quantitatively we conducted a leave one out analysis. In particular we constructed leave one out alignments by deleting one target site sequence from an input alignment. Then, for each alignment in which the target sequence *w *was left out, we computed a *SBM *set and determined the score *S*(*w*) of the target site that was left out. If *S*(*w*) < 1, the threshold needs to be adjusted to *S*_min _= *S*(*w*) in order to detect *w*. We therefore scanned the respective UTR set with *S*_min _= min {1, *S*(*w*)} and determined the number of predicted targets.

An input alignment of *n *sequences allows construction of *n *- 1 leave one out alignments (we did not leave out the reverse complement of the miRNA), so data sets containing more experimentally validated target sites clearly result in more meaningful leave one out analyses. We therefore chose the four miRNAs that had the greatest number of known experimentally validated targets; *D. melanogaster miR-7 *and *C. elegans let-7*, which both targeted 15 unique UTR regions as well as *D. melanogaster miR-4 *(8 unique targets) and *C. elegans miR-84 *(7 unique targets). In total 2,484,850 UTR regions were scanned in the *C. elegans *set compared with to 4,409,641 regions in the *D. melanogaster *set. The score of each left out target along with the number of regions with a score equal to or greater than this value in the scan using the full alignment are shown in Table 3.

**Table 3:** Leave one out analysis

***Drosophila melanogaster*, miR-7**		
**Target**	**LOO score**	⩾ **LOO score**

CG12487.3/223–241	0.946	94
CG5185.3/279–297	1.000	34
CG3096.3/152–170	1.000	34
CG12487.3/250–268	1.000	34
CG3166.3/1100–1118	0.951	76
CG6096.3/103–121	1.000	34
CG8346.3/78–96	0.966	58
CG5185.3/334–352	1.000	34
CG6494.3/447–465	0.919	155
CG6096.3/24–42	1.000	34
CG6096.3/68–86	0.961	65
CG8328.3/63–81	0.773	2015
CG3166.3/1586–1602	0.855	393
CG3166.3/29–46	0.845	513
CG3166.3/1294–1312	0.861	521

***Caenorhabditis elegans*, let-7**		

**Target**	**LOO score**	⩾ **LOO score**

ZK792.6/247–264	0.959	3561
F38A6.1a/271–288	1.000	1708
C18D1.1.1/526–542	0.906	10458
ZK792.6/666–683	0.959	3522
ZK792.6/458–475	0.929	7311
F38A6.1a/133–150	0.874	19177
C01G8.9a/21–38	0.850	23906
ZK792.6/132–148	0.859	20570
C01G8.9a/159–175	0.813	30895
ZK792.6/190–207	0.807	41812
C12C8.3a/693–709	0.791	39369
C12C8.3a/742–757	1.000	1499
ZK792.6/484–499	0.898	10232
F11A1.3a/1007–1021	0.948	4658
ZK792.6/343–361	0.955	4352

***Drosophila melanogaster*, miR-4**		

**Target**	**LOO score**	⩾ **LOO score**

CG6096.3/135–154	0.755	3118
CG8328.3/27–45	1.000	8
CG3096.3/33–52	0.929	161
CG3096.3/138–157	0.877	473
CG5185.3/46–65	0.960	64
CG12487.3/188–208	0.820	1298
CG12487.3/62–82	0.871	627
CG6096.3/210–230	0.908	207

***Caenorhabditis elegans*, miR-84**		

**Target**	**LOO score**	⩾ **LOO score**

ZK792.6/126–148	0.804	4970
ZK792.6/187–207	0.552	132626
ZK792.6/249–264	0.947	355
ZK792.6/342–361	0.761	12552
ZK792.6/460–475	0.858	2012
ZK792.6/479–499	0.739	18375
ZK792.6/665–683	0.726	15846

"target" gives validated target sequence accession/start-end; "miRNA" gives miRNA targeting that region; "⩾ LOO score" gives mean number of regions scoring equal to or greater than the left out sequence.

The *SBM *method appears to show a greater degree of accuracy in the *D. melanogaster *miR-7 results. Here the mean score of the left out target is 0.9385 and the mean number of target regions scoring greater than or equal to the left out sequence is 273 (0.006% of the total UTR regions scanned). The *C. elegans *let-7 scan indicates a lower degree of specificity, with an average score of 0.9032, returning a mean of 14869 regions with a score greater than or equal to the score of the left out validated target sequence. This represents 0.598% of the sequence database that was searched. For *D. melanogaster *miR-4 the *SBM *method gave a mean score of 0.890 with an average of 745 target regions scoring greater than or equal to the left out sequence (0.017% of the total UTR regions scanned), and for *C. elegans *miR-84 a mean score of 0.770 was obtained and an average of 26677 target regions scoring greater than or equal to the left out sequence was returned (1.074% of the total UTR regions scanned). The decrease in specificity in the *C. elegans *miR-84 results is largely due to a single leave one out test in which over 132626 sequences scored higher than the left out sequence (which received a score of 0.552).

Overall, the lowering of the threshold required to detect a word not in the input set results in a moderate increase in the number of reported hits, which is indicative of a high specificity even with the reduced threshold.

In order to assess the performance of the algorithm when few known targets are provided in the input alignment were-ran the *C. elegans let-7 *and *D. melanogaster miR-7 *scans but this time split each of the alignments of 15 validated targets into two subalignments containing 8 and 7 sequences respectively. Table 4 shows that as the number of sequences used to build the *SBM *decreases, so does the mean score of the left out sequences. This indicates, as might be expected that as the number of sequences left out of the alignment increases the specificity decreases.

**Table 4:** Leave several out analysis

	**15 targets**	**14 targets**	**8 targets**	**7 targets**
Mean score *C. elegans let-7*	1.000	0.903	0.851	0.810
Mean number returned *C. elegans let-7*	1708	14869	18032	17225
Mean score *D. melanogaster miR-7*	1.000	0.938	0.908	0.890
Mean number returned *D. melanogaster miR-7*	28	273	509	138

Shows mean scores and mean number of regions scoring above maximal consistent threshold for alignments containing 15, 14, 8 and 7 validated targets.

### Comparison with miRanda

We also compared the performance of the *SBM *method with miRanda v1.9, a commonly used target prediction tool [10]. miRanda takes a single miRNA sequence as input and searches a sequence dataset for potential target regions. It uses two different criteria to detect potential target sites, the alignment score and the MFE of the miRNA bound to the potential target sequence.

In order to obtain results with miRanda that could be meaningfully compared with the *SBM *method, we used miRanda to score every potential target site across each of the UTR sequences. To do this we split each of the UTRs into 30 nt sequence windows covering the entire length of each UTR and used this as our sequence database for the miRanda scan. Since the same target region may be scored more than once using this approach, we removed any duplicate regions from the results before the comparison. By default miRanda uses relatively stringent threshold values which do not necessarily recover all known target regions, i.e. classification is not consistent. For this reason miRanda was run using a negative score threshold and a positive energy threshold which allowed us to obtain a wide distribution of scores and to ensure consistency.

Table 5 provides an overview of the miRanda comparison, the full results can be found in the supplementary materials [see Additional file 1]. In general the *SBM *method compared favourably with miRanda. This is not unexpected as we incorporate additional information into our searches. For example the *cel-let7 *results show that an average of 14869 regions had a score that was at least as high as the left out sequence using *SBM *whereas an average of 92332 regions scored at least as high as the validated target using miRanda. This difference was more pronounced in the *dme-miR-7 *results where an average of 273 sequences scored equal to or better than the left out sequences and an average of 8868 sequences scored at least as high as the validated target using miRanda. The *SBM *method returned an average of 745 sequences scoring equal to or better than the left out sequence for *dme-miR-4 *in comparison to an average of 11488 sequences that scored at least as high as the validated target using miRanda. An average of 26677 target regions were returned using the *SBM *method for *cel-miR-84 *compared with 190693 using miRanda.

**Table 5:** Summary of results for the leave one out analysis

**miRNA**	**LOO score**	⩾ **LOO score**	**miRanda(s)**	⩾ **miRanda(s)**	**miRanda(e)**	⩾ **miRanda(e)**	⩾ **miRanda(se)**
cel-let-7	0.903	14869	119	92332	-15.46	60266	23992
cel-miR-84	0.770	26677	106	190693	-10.19	150137	48538
dme-miR-7	0.938	273	159	8868	-21.69	7227	2129
dme-miR-4	0.890	745	131	11488	-8.51	184134	5325

"miRNA" gives miRBase accession of the miRNA sequence; "LOO score" gives mean score of the targets left out of the *SBM*; "⩾ LOO score" gives mean number of regions scoring equal to or greater than the left out sequence; "miRanda(s)" gives raw score of the miRanda hit of lowest scoring target region; "⩾ miRanda(s)" gives number of regions with returned using the maximal consistent score threshold; "miRanda(e)" gives minimum free energy (MFE) of the miRanda hit of the least stable target region; "⩾ miRanda(e)" gives number of regions with returned using the maximal consistent MFE threshold; "⩾ miRanda(se)" gives number of regions with returned using the maximal consistent combined score and MFE threshold.

We determined the maximal consistent threshold for miRanda results by filtering out all candidates with an alignment score lower than the lowest scoring validated target. The remaining candidates are then filtered further by removing any sequence with an MFE of greater than the MFE of the highest (least stable) of the validated targets. The number of regions returned using the maximal consistent threshold in miRanda were 23992 for *cel-let7 *in contrast to the 1708 returned using the *SBM *method with maximal consistent threshold. 48538 regions were recovered for *cel-miR-84 *compared with 123 using *SBM*, 2129 for *dme-miR-4 *in comparison to 23 with *SBM *and 5325 for *dme-miR-7*, with the *SBM *method returning 28.

## Discussion

We have presented a new method, *SBM*, that allows the use of miRNA target site sequences in addition to the miRNA sequence itself to search for novel target sites. We have demonstrated its application to target prediction for a variety of miRNA examples from different organisms and have shown that it performs well in comparison to miRanda. Many computational methods for target prediction tend to suffer from a lack of specificity [15]. The *SBM *method allows the use of all known target sequences in the search, and is designed to provide maximum specificity whilst recovering all members present in the starting alignment. Thus, as the number of experimentally validated miRNA targets grows, the *SBM *method should provide an attractive addition to the available miRNA target site detection methods.

Many current target prediction techniques are based on algorithms with fixed parameters (such as base pairing rules or binding energies) that are used to assess potential targets by matching them to the miRNA sequence. These algorithms are designed to reflect molecular target recognition mechanisms that are assumed to apply to miRNA target recognition in general. Tailoring these algorithms to reflect mechanisms that are specific to the miRNA is difficult or impossible. In contrast to this, the *SBM *method can capture aspects of specific binding mechanisms by extracting such specific information from the set of validated target site sequences. This also makes the method generic in that it can be applied to any organism without having to assume any prior knowledge of specific target recognition mechanisms.

Due to the small number of validated targets for each miRNA, the maximal consistent threshold used in the *SBM *method is rather stringent. We chose this threshold to facilitate comparison of the method to miRanda. For many applications lowering thresholds to increase sensitivity at the cost of losing some specificity may be advisable. The specificity advantage of the *SBM *method can be expected to be partly independent of the threshold, since moderate relaxation of the threshold for a classifier that attains a high level of specificity with a given threshold can be assumed to retain some of the specificity advantage.

As with all scoring matrix approaches, the *SBM *method is limited by the quality of the input data. Firstly if a false positive target sequence is provided as input the method will be adversely affected, therefore only experimentally validated targets should generally be used as input. Secondly the quality of the input alignment is extremely important and a poor quality alignment will lead to poor performance. miRNAs are relatively short (~21 nt) which means that in many cases they can be aligned quite accurately using multiple alignment algorithms such as ClustalW [25] and MUSCLE [31]. In some cases however, the conservation between sites targeted by the same miRNA is very low, meaning that an accurate sequence alignment is hard to produce using automated methods. In such cases it may be favourable to hand curate alignments in order to ensure quality and obtain optimal *SBM *results. Thirdly, although the short length of miRNAs also allows for the integration of gapped alignments in the *SBM *method, the method will only search for the gap patterns contained in the input alignment. Thus, if targets contain insertion/deletion patterns which are not specified in this way, then they may receive a lower score or even be missed completely depending on the threshold used in the search.

Several miRNA target prediction systems have implemented post-processing steps in order to increase their specificity. The most commonly used filtering approach is to look for cross-species conservation of target sites. Here target sites that appear not to be conserved between multiple species are filtered out from the search results, removing false positives, and leading to increased specificity. This type of approach could be applied to results obtained with *SBM *to further increase the specificity of target predictions. However, we note that this might also lead to a reduction in sensitivity as it is now known that miRNAs themselves are not always conserved between related species (e.g. [32]). Another possibility is to post-process based on target site accessibility. It has recently been shown that taking into account target site accessibility in the 3' UTR can improve target prediction accuracy [33]. For instance if a predicted target site is part of a stable secondary structure (and is therefore already involved in base-pairing) it is less likely that the miRNA will be able to bind to the target causing the translational repression of the mRNA. In conclusion, we have presented a promising new method for miRNA target prediction, *SBM*, that employs a generic scoring matrix approach and incorporates experimentally validated targets. Since the number of validated targets is constantly growing, *SBM *should provide a useful new addition to the current target prediction toolbox.

## Availability and Requirements

The code, together with documentation and examples, is freely available for download from .

## Supplementary Material

Additional file 3 Raw result files. Raw *SBM *results files, alignments of miRNA targets used in the analysis and a list of overlapping targets predicted by both *SBM *and miRanda. Click here for file

Additional file 1 tableS1 – *SBM *comparison with miRanda. Full results of the *SBM *comparison with miRanda for each of the four miRNAs tested. Click here for file

Additional file 2 tableS2 – Number of overlapping predictions. Summary table showing the number of target regions predicted by *SBM *and miRanda using default parameters and their overlap. Click here for file
