# Supplementary material for: A scoring matrix approach to detecting miRNA target sites
Source: Algorithms Mol Biol. 2008 Mar 31;3:3. doi: 10.1186/1748-7188-3-3 (PMC2365947; doi:10.1186/1748-7188-3-3)
Supplement: Additional file 1 — tableS1 – SBM comparison with miRanda. Full results of the SBM comparison with miRanda for each of the four miRNAs tested. [file 1748-7188-3-3-S1.pdf]

Target	LOO score	⩾ LOO score	miRanda(s)	⩾ miRanda(s)	miRanda(e)	⩾ miRanda(e)	⩾ miRanda(se)
ZK792.6/247-264	0.9587762	3561	109	83402	-14.10	38347	19155
F38A6.1a/271-288	1.0000000	1708	92	390274	-10.15	273523	159046
C18D1.1.1/526-542	0.9064860	10458	123	25838	-11.21	170342	19261
ZK792.6/666-683	0.9588374	3522	120	37964	-14.88	24456	8034
ZK792.6/458-475	0.9286717	7311	127	15875	-13.18	63901	9280
F38A6.1a/133-150	0.8742177	19177	108	95809	-14.56	29677	16710
C01G8.9a/21-38	0.8496634	23906	123	25838	-15.23	19926	5901
ZK792.6/132-148	0.8591035	20570	112	66769	-12.00	117457	33464
C01G8.9a/159-175	0.8134508	30895	113	61153	-13.33	58770	21054
ZK792.6/190-207	0.8068349	41812	91	403453	-13.33	58770	46851
C12C8.3a/693-709	0.7908144	39369	151	888	-25.13	14	9
C12C8.3a/742-757	1.0000000	1499	163	84	-25.06	16	4
ZK792.6/484-499	0.8981500	10232	107	102609	-17.86	3488	2822
F11A1.3a/1007-1021	0.9483082	4658	128	13871	-17.91	3345	1340
ZK792.6/343-361	0.9552215	4352	113	61153	-13.94	41961	16950
**Mean**	0.9032357	14869	119	92332	-15.46	60266	23992

Table 1: Summary of the results for let-7 target predictions in Caenorhabditis elegans. Column "target" lists accession of the location of the validated target (UTR accession, start and end position), column "LOO score" shows the score for that target when left out of the *SBM*, column "⩾ LOO score" shows the number of regions scoring equal to or greater than the left out sequence, column "miRanda(s)" shows the raw score of the miRanda hit corresponding to the target region, column "⩾ miRanda(s)" shows the number of regions scoring equal to or greater than the target region, column "miRanda(e)" shows the minimum free energy (MFE) of the miRanda hit corresponding to the target region, column "⩾ miRanda(e)" shows the number of regions with an equal or more stable MFE than the target region, column "⩾ miRanda(se)" shows the number of regions with an equal or more stable MFE and a score greater than or equal to the target region.

Target	LOO score	⩾ LOO score	miRanda(s)	⩾ miRanda(s)	miRanda(e)	⩾ miRanda(e)	⩾ miRanda(se)
ZK792.6/126-148	0.8042310	4970	103	149244	-7.50	432158	114090
ZK792.6/187-207	0.5515072	132626	84	612304	-10.95	74633	61242
ZK792.6/249-264	0.9471851	355	114	55099	-10.61	91546	23993
ZK792.6/342-361	0.7607794	12552	102	160281	-8.80	235444	88454
ZK792.6/460-475	0.8575555	2012	126	15024	-10.20	114702	12066
ZK792.6/479-499	0.7394901	18375	94	317848	-12.08	38304	27872
ZK792.6/665-683	0.7264934	15846	122	25051	-11.22	64174	12052
**Mean**	0.7696059	26677	106	190693	-10.19	150137	48538

Table 2: Summary of the results for miR-84 target predictions in Caenorhabditis elegans. Column "target" lists accession of the location of the validated target (UTR accession, start and end position), column "LOO score" shows the score for that target when left out of the *SBM*, column "⩾ LOO score" shows the number of regions scoring equal to or greater than the left out sequence, column "miRanda(s)" shows the raw score of the miRanda hit corresponding to the target region, column "⩾ miRanda(s)" shows the number of regions scoring equal to or greater than the target region, column "miRanda(e)" shows the minimum free energy (MFE) of the miRanda hit corresponding to the target region, column "⩾ miRanda(e)" shows the number of regions with an equal or more stable MFE than the target region, column "⩾ miRanda(se)" shows the number of regions with an equal or more stable MFE and a score greater than or equal to the target region.

Target	LOO score	⩾ LOO score	miRanda(s)	⩾ miRanda(s)	miRanda(e)	⩾ miRanda(e)	⩾ miRanda(se)
CG12487.3/223-241	0.9464120	94	164	215	-22.76	127	44
CG5185.3/279-297	1.0000000	34	173	36	-24.25	45	10
CG3096.3/152-170	1.0000000	34	168	117	-24.14	46	16
CG12487.3/250-268	1.0000000	34	179	8	-24.71	38	6
CG3166.3/1100-1118	0.9505960	76	140	3490	-18.77	2322	621
CG6096.3/103-121	1.0000000	34	172	47	-23.80	63	12
CG8346.3/78-96	0.9659194	58	185	2	-28.03	2	1
CG5185.3/334-352	1.0000000	34	170	83	-23.24	93	25
CG6494.3/447-465	0.9188494	155	179	8	-25.24	25	3
CG6096.3/24-42	1.0000000	34	171	64	-23.71	72	19
CG6096.3/68-86	0.9614793	65	170	83	-23.71	72	21
CG8328.3/63-81	0.7726001	2015	145	2210	-16.48	10689	1001
CG3166.3/1586-1602	0.8547854	393	138	4162	-16.44	10990	1630
CG3166.3/29-46	0.8454907	513	130	11501	-16.84	8407	2226
CG3166.3/1294-1312	0.8607346	521	108	111001	-13.26	75409	26300
**Mean**	0.9384578	273	159	8868	-21.69	7227	2129

Table 3: Summary of the results for mir-7 target predictions in Drosophila melanogaster. Column “target” lists accession of the location of the validated target (UTR accession, start and end position), column “LOO score” shows the score for that target when left out of the *SBM*, column “⩾ LOO score” shows the number of regions scoring equal to or greater than the left out sequence, column “miRanda(s)” shows the raw score of the miRanda hit corresponding to the target region, column “⩾ miRanda(s)” shows the number of regions scoring equal to or greater than the target region, column “miRanda(e)” shows the minimum free energy (MFE) of the miRanda hit corresponding to the target region, column “⩾ miRanda(e)” shows the number of regions with an equal or more stable MFE than the target region, column “⩾ miRanda(se)” shows the number of regions with an equal or more stable MFE and a score greater than or equal to the target region.

Target	LOO score	⩾ LOO score	miRanda(s)	⩾ miRanda(s)	miRanda(e)	⩾ miRanda(e)	⩾ miRanda(se)
CG6096.3/135-154	0.7550454	3118	143	735	-11.44	26630	491
CG8328.3/27-45	1.0000000	8	135	3154	-8.69	128342	2427
CG3096.3/33-52	0.9287263	161	130	6986	-7.00	305330	5993
CG3096.3/138-157	0.8767518	473	136	2730	-7.30	264578	2638
CG5185.3/46-65	0.9598055	64	139	1586	-8.84	118531	1390
CG12487.3/188-208	0.8200697	1298	112	56515	-8.54	138880	18111
CG12487.3/62-82	0.8714641	627	127	10317	-10.03	61435	3372
CG6096.3/210-230	0.9076755	207	128	9883	-6.26	429349	8181
**Mean**	0.8899422	745	131	11488	-8.51	184134	5325

Table 4: Summary of the results for mir-4 target predictions in Drosophila melanogaster. Column “target” lists accession of the location of the validated target (UTR accession, start and end position), column “LOO score” shows the score for that target when left out of the *SBM*, column “⩾ LOO score” shows the number of regions scoring equal to or greater than the left out sequence, column “miRanda(s)” shows the raw score of the miRanda hit corresponding to the target region, column “⩾ miRanda(s)” shows the number of regions scoring equal to or greater than the target region, column “miRanda(e)” shows the minimum free energy (MFE) of the miRanda hit corresponding to the target region, column “⩾ miRanda(e)” shows the number of regions with an equal or more stable MFE than the target region, column “⩾ miRanda(se)” shows the number of regions with an equal or more stable MFE and a score greater than or equal to the target region.
