# Supplementary material for: A scoring matrix approach to detecting miRNA target sites
Source: Algorithms Mol Biol. 2008 Mar 31;3:3. doi: 10.1186/1748-7188-3-3 (PMC2365947; doi:10.1186/1748-7188-3-3)
Supplement: Additional file 2 — tableS2 – Number of overlapping predictions. Summary table showing the number of target regions predicted by SBM and miRanda using default parameters and their overlap. [file 1748-7188-3-3-S2.pdf]

Organism	miRNA	*SBM*	miRanda	Overlap
C. elegans	*cel-miR-273*	2	661	0
C. elegans	*cel-let-7*	1708	210	33
C. elegans	*cel-miR-84*	123	33	0
D. melanogaster	*dme-miR-11*	4	369	0
D. melanogaster	*dme-miR-2*	4	755	2
D. melanogaster	*dme-miR-4*	31	14	0
D. melanogaster	*dme-miR-7*	43	373	17
M. musculus	*mmu-miR-124*	3	4645	0
M. musculus	*mmu-miR-206*	3	5811	1
H. sapiens	*hsa-miR-1*	3	171	0
H. sapiens	*hsa-miR-122*	3	11228	2

Table 1: Number of targets predicted by *SBM* and miRanda and number of overlaps between the two methods column "miRNA" lists the miRBase accession of the miRNA tested, column "*SBM*" shows the number of targets predicted by *SBM* with default threshold (-t 1), column "miRanda" shows the number of targets predicted by miRanda using default settings (no parameters given), column "Overlap" shows the number of targets that were predicted by both methods.
